# Human Adipose Tissue Derived Extracellular Matrix and Methylcellulose Hydrogels Augments and Regenerates the Paralyzed Vocal Fold

**DOI:** 10.1371/journal.pone.0165265

**Published:** 2016-10-21

**Authors:** Dong Wook Kim, Eun Ji Kim, Eun Na Kim, Myung Whun Sung, Tack-Kyun Kwon, Yong Woo Cho, Seong Keun Kwon

**Affiliations:** 1 Department of Otorhinolaryngology-Head and Neck Surgery, Seoul National University Hospital, Seoul National University College of Medicine, Seoul, Republic of Korea; 2 Department of Chemical Engineering, Hanyang University, Ansan, Gyeonggi-do 426–791, Republic of Korea; 3 Department of Pathology, Asan Medical Center, University of Ulsan College of Medicine, Seoul, Republic of Korea; Kyoto Daigaku, JAPAN

## Abstract

Vocal fold paralysis results from various etiologies and can induce voice changes, swallowing complications, and issues with aspiration. Vocal fold paralysis is typically managed using injection laryngoplasty with fat or synthetic polymers. Injection with autologous fat has shown excellent biocompatibility. However, it has several disadvantages such as unpredictable resorption rate, morbidities associated with liposuction procedure which has to be done in operating room under general anesthesia. Human adipose-derived extracellular matrix (ECM) grafts have been reported to form new adipose tissue and have greater biostability than autologous fat graft. Here, we present an injectable hydrogel that is constructed from adipose tissue derived soluble extracellular matrix (sECM) and methylcellulose (MC) for use in vocal fold augmentation. Human sECM derived from adipose tissue was extracted using two major steps—ECM was isolated from human adipose tissue and was subsequently solubilized. Injectable sECM/MC hydrogels were prepared by blending of sECM and MC. Sustained vocal fold augmentation and symmetric vocal fold vibration were accomplished by the sECM/MC hydrogel in paralyzed vocal fold which were confirmed by laryngoscope, histology and a high-speed imaging system. There were increased number of collagen fibers and fatty granules at the injection site without significant inflammation or fibrosis. Overall, these results indicate that the sECM/MC hydrogel can enhance vocal function in paralyzed vocal folds without early resorption and has potential as a promising material for injection laryngoplasty for stable vocal fold augmentation which can overcome the shortcomings of autologous fat such as unpredictable duration and morbidity associated with the fat harvest.

## Introduction

Glottal insufficiency, or vocal fold paralysis, results from various etiologies and can induce voice changes, swallowing difficulties, and issues with aspiration. This medical condition is usually treated using injection laryngoplasty.[1] Based on clinical experience and studies related to glottal insufficiency, an ideal material for injection laryngoplasty should have the following characteristics. It should be easily injectable into the vocal folds in an outpatient setting. It should sufficiently restore the volume of the atrophied glottis. It should medialize the vocal fold permanently, be highly biocompatible and not induce an inflammatory response. However, none of the currently used materials for injection laryngoplasty possess all of these characteristics.

Calcium hydroxyapatite (CaHA) is the only injection material approved by the FDA for laryngoplasty, and it has been commonly used because of its lower cost, morbidity, and invasiveness compared with those of conventional open thyroid framework surgery.[2] However, CaHA cannot maintain its augmentation effect long term, and it has been reported to induce various complications such as inflammation, granulation, migration, and inhibition of vocal fold vibration.[3]

Autologous fat injection is considered to be durable and long-lasting, with excellent biocompatibility. Several studies have reported that autologous fat injection has long-lasting effects that are comparable to the functional outcomes seen in framework surgery such as medialization thyroplasty.[4–8] However, unlike injection laryngoplasty using CaHA, which can be easily performed in an outpatient clinic, autologous fat injection laryngoplasty requires a liposuction procedure that needs to be performed under general anesthesia in an operating room. Injection laryngoplasty with fat requires large gauge needles, such as 18 gauge, which makes it difficult to localize the injected material precisely into the muscle layer of vocal fold. Another concern with fat injection is the possibility of unpredictable early resorption. The efficacy of fat injection is often doubted due to variable resorption rates and unpredictability in postoperative outcomes. The resorption rate of grafted fat tissue ranges from 20% to 90%.[9–11] It was reported that injected fat was absorbed within 1 month and was completely resorbed within 5 months.[12, 13] To prevent early resorption, lipoinjection into vocal folds is performed such that there is substantial over injection of the volume. However, this excessive fatty tissue can induce poor voice quality and respiratory distress.

To overcome these limitations, we developed an easily injectable, soluble extracellular matrix (sECM) and methylcellulose (MC) hydrogel for use in injection laryngoplasty. The sECM/MC solution exhibited thermosensitive sol-to-gel transition, remaining a viscous liquid at 4°C and forming a translucent hydrogel at 37°C. Human adipose tissue, the most prevalent and expendable tissue in the body, can be harvested in large quantities with minimal morbidity and has received much attention as a rich source of ECM. Numerous studies have reported on the biological effects of ECM-based three-dimensional (3-D) tissue engineering scaffolds for use in regenerative medicine.[14–18] Human adipose-derived ECM grafts have greater biostability than autologous fat graft.[16, 17] ECM scaffolds derived from human adipose tissue have been reported to increase graft volume and form new adipose tissue with numerous blood vessels.[14–18] Here, we introduce sECM hydrogel as a novel material for injection laryngoplasty and present preliminary animal study data focusing on the effects of stable augmentation and its biocompatibility.

## Materials and Methods

### Materials

All experiments performed in this study were approved by the Institutional Animal Care and Use Committee of Seoul National University Hospital (approval number: 14-0005-S1A0) and performed in accordance with the ethical guidelines of the committee. All efforts were made to minimize suffering.

### Preparation of sECM/MC hydrogel

The sECM was prepared from human adipose tissue as previously described.[14–18] Briefly, human adipose tissue was obtained with informed consent as approved by the Institutional Review Board of the Catholic University of Korea, College of Medicine (KIRB-00419-023). The adipose tissue was washed several times with distilled water to remove blood components. Distilled water was added to the adipose tissue and the tissue/water (1:1) mixture was homogenized for 3 min using a commercial blender. The suspension was centrifuged at 16,500 xg for 5 min at 4°C to remove oil components. For solubilization of ECM, the extracted solid ECM was washed with 3.4 M NaCl, centrifuged at 20,000 xg for 30 min at 4°C and suspended in 4 M urea buffer containing protease inhibitor cocktail for 12 h at 4°C. The mixture was centrifuged at 20,000 xg for 60 min at 4°C to remove insoluble materials. The residues were re-extracted in 4 M guanidine containing protease inhibitor cocktail for 12 h at 4°C, and were centrifuged. The supernatant was dialyzed extensively in dialysis tubing against 30 volumes of Tris-buffered saline (TBS) for 24 h at 4°C, concentrated using a 3,000 MWCO Amicon Ultra-15 centrifugal filter device, and the final sECM was lyophilized. The lyophilized sECM powders and MC powders were sterilized by ethylene oxide (EO) gas.

The sECM solution (30% w/v) was prepared by dissolving 3 g of lyophilized sECM in 10 mL of phosphate buffered saline (PBS) for 24 h at 4°C. MC solution (7.5% w/v) was prepared using dispersion. Briefly, 0.75 g of MC (viscosity of 15 cP) was thoroughly wetted in 10 mL of PBS and incubated at 90°C for 60 min. Then, the solution was equilibrated overnight at 4°C. Subsequently, sECM and MC solutions were combined at a volume ratio of 1:4 to a final concentration of 6% sECM and 6% MC, and the mixture was stirred to homogeneity at 4°C. The sECM/MC solution was stored at 4°C until use.

### Recurrent laryngeal nerve (RLN) section, sECM/MC hydrogel injection laryngoplasty, and group classification

Twenty five male New Zealand white rabbits (Koatech Laboratory Animal Company, Korea) weighing 3.0 to 3.5 kg were obtained. Five animals were used as normal control. Unilateral recurrent laryngeal nerve section to induce vocal fold palsy was performed in twenty animal as previously described.[19] Zoletil (50 mg/kg) was administered intramuscularly for anesthesia and subcutaneous fat and strap muscles were dissected at the midline through a 2.5 cm vertical incision made at the level of the cricoid. After thyroid isthmectomy, the left thyroid lobe was laterally reflected, and the inferior thyroid vessel was identified at the medial surface of the thyroid gland. The left RLN was identified parallel to the inferior thyroid vessels. The RLN was severed for approximately 2 cm to prevent spontaneous re-anastomosis. Subcutaneous tissue and skin were closed with a surgical stapler. Immediately after the procedure, laryngeal endoscopic exam was performed to confirm unilateral vocal fold palsy using a 4.0 mm 30 degree rigid endoscope (Richards, Knittlingen, Germany). Prophylactic antibiotics (cephazolin) were administrated intramuscularly for 3 days.

One week after RLN sectioning, rabbits were randomly divided into 4 equal groups of 5 rabbits using a blocked randomization method. Groups 1, 2, and 3 received sECM/MC hydrogel injection laryngoplasty and group 4 acted as the control group with no injection procedure. The sECM/MC hydrogel injection was conducted under anesthesia with intramuscular injection of Zoletil (50 mg/kg). Each injection (0.1 cc/rabbit) was administered using a syringe with a 23 G spinal needle under the guidance of a 4.0 mm 30 degree rigid endoscope. The injection needle was placed laterally at the tip of the vocal process such that the vocal process could rotate medially. For the following 3 days, rabbits were given prophylactic antibiotics. All animals were monitored daily for weight, coughing, sputum production, wheezing, and dyspnea.

**Fig 1:**
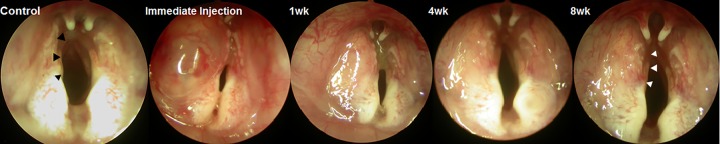
Laryngoscopic images after injection laryngoplasty of sECM/MC hydrogels into the left paralyzed vocal fold. The sECM/MC hydrogel injection group exhibited a straight and medialized vocal fold (white arrowhead) while the control group had a curved and lateralized vocal fold (black arrowhead), which is likely due to denervation of the recurrent laryngeal nerve.

For histological studies and functional analyses, groups 1, 2, and 3 were sacrificed at 1, 4, and 8 weeks after sECM/MC hydrogel injection laryngoplasty, respectively. From group 4, two rabbits were randomly selected for sacrifice at 4 weeks after sECM/MC hydrogel injection laryngoplasty and the remaining rabbits were sacrificed at 8 weeks. All groups received standard laryngoscopic examination using a 4.0 mm 30 degree rigid endoscope immediately post injection laryngoplasty and at 1, 4, and 8 weeks after sECM/MC hydrogel injection laryngoplasty. Images were taken of vocal folds with a digital camera (E4500, Nikon, Tokyo, Japan) that was attached to the endoscope (Fig 1).

### Assessment of vocal fold vibration for functional analysis

Group 3 and control group animals were humanely sacrificed, and the larynges were removed through total laryngectomy. Vocal fold vibrations were examined using a high-speed camera as previously described.[19, 20] To better visualize the vocal fold, the superior portion of the thyroid cartilage and supraglottic structures were removed. The arytenoid cartilage was sutured using a Prolene 6–0 suture to close both vocal folds. The trimmed larynx was mounted, and humidified air was injected into the larynx to generate vocal fold vibration. Vocal fold vibration during induced phonation was imaged using MotionXtra NR4S2 high-speed video camera (DEL Imaging Systems, Cheshire, Connecticut, USA). High-speed video data were recorded at 8,000 images per sec with a spatial resolution of 256 horizontal × 512 vertical pixels. Illumination was provided by a 300-W xenon light source.

**Fig 2:**
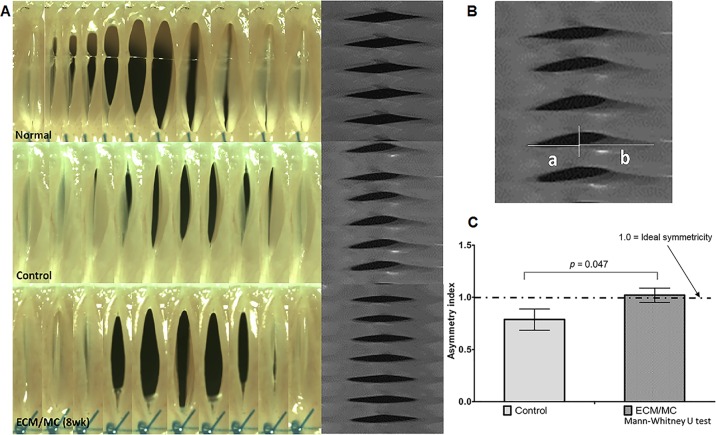
**Representative serial images of high-speed camera recording at 8 weeks (A), the asymmetric index using videokymograms (B) and the results of asymmetry index for vocal functional analysis (C)**. (A) Normal and symmetrical vocal contacts showed no change in the vibration of vocal mucosa in sECM/MC groups relative to the control group. (B) The maximum distance in the left denervated vocal fold (*a*) was compared to the right vocal fold (*b*) using a videokymogram to generate an asymmetry index. The asymmetry index was calculated as follows: Asymmetry index = *a* / *b*. (C) The mean asymmetry index of the sECM/MC hydrogel group (1.020 ± 0.069) and the control group (0.787 ± 0.102) are shown (*p* = 0.047 using a Mann-Whitney U test). In diseased conditions, the index deviates from the value of 1.0

The maximum amplitude of the vocal wave was measured to evaluate the symmetricity of the vocal mucosal vibration using videokymograms that were obtained from the high speed images using Metamorph^®^ (Molecular Device, Sunnyvale, CA) and *Image J* imaging software (National Institute of Mental Health, Bethesda, MD).[21] The maximum distance from the midline of the glottis to the free edge of the vocal fold was measured at the mid antero-posterior portion of the vocal fold using the maximum open phase of a videokymogram (Fig 2A). The maximum distance of the left denervated vocal fold (*a*) was divided by the maximum distance of the right vocal fold (*b*) to create a ratio that was referred to as the asymmetry index. The asymmetry index is calculated as follows (Fig 2B):

Asymmetry index=a/b

The asymmety index shows a degree of the symmetricity in bilateral larynx. A physiologic phonation of normal mammals (dog, rabbit and human, etc.) is achieved by the symmetrical vibration of bilateral larynxes. Therefore, the asymmetry index of healthily vibratory larynx should be expected to be near the value of 1.0. In diseased conditions, including vocal paralysis or severe scar formation at vocal mucosa, the index deviates from the value of 1.0.[22]

### Histological analysis and volume calculations of the injected sECM/MC hydrogel

Specimens from groups 1, 2, 3 and 4 were fixed for 24 h in 10% formalin, and were serially sliced at 4 μm thick using a microtome in the axial plane from the false vocal fold to the level of the subglottis. Standard hematoxylin and eosin (H&E) staining was performed to assess the amount of remaining injection material and determine the presence of any inflammatory tissue reactions. For collagen, hyaluronic acid, and elastin fiber staining, sections were stained with Masson’s trichrome (MT), Alcian blue (AB), and Verhoeff-Van Gieson (VVG), respectively. In the key section of MT histology, quantitative analysis of collagen fiber deposition within the area of sECM/MC hydrogels injection was performed using Metamorph^®^ software to calculate the total area of staining. The mean area percentage (%) of collagen fiber in the cross-sectional area of the injection material was analyzed according to the experimental period. In addition, an immunofluorescence assay for collagen type I was performed to evaluate fibrosis. Tissue sections were deparaffinized, incubated with a hot stirrer for 10 min in citrate buffer (Invitrogen, Frederick, USA, 005000). Samples were then incubated at room temperature for 30 min followed by 0.5% Triton X-100 solution for 15 min. Samples were blocked using 3% BSA in PBS for 1 h at room temperature, and primary antibody (Collagen type I, Abcam, Bristol, UK, ab6308) was incubated at 4°C overnight. After washing with PBS, secondary antibody (Invitrogen, Frederick, USA, Alexa Goat-594/Alexa mouse-488) was incubated at room temperature for 1 h, and nuclei were stained with DAPI (Molecular Probes, Oregon, USA, H3570). Imaging was performed on a fluorescent microscope. Immunohistochemistry (IHC) using monoclonal mouse anti-rabbit macrophage clone RAM11 (Dako, Ely, UK, M0633) was also performed for confirmation of foamy histiocyte. An experimentally blinded pathologist (E.N.K) reviewed the samples.

The volume of remaining material was calculated using a modified procedure of a previously reported method.[23] For each time point, the area of remaining injected material was measured in pixels from 10 consecutive H&E-stained slides (each 100 μm interval) using computerized image analysis software (Leica Q win V3, Konvision Corporation, Seoul, South Korea).[19] The volume of the remaining sECM/MC hydrogel was estimated using the following formula:

Total volume=the sum of cross-sectional material areas from10consecutive slides×100μm

### Statistical analysis

Experimental data were expressed as median with interquartile ranges. Non-parametric statistical test using SPSS 21.0 statistical software (SPSS) was performed, and statistical significance was recognized as *p* < 0.05.

## Results

The sECM/MC hydrogel was readily injected into the paralyzed vocal folds of all rabbits for groups 1, 2, and 3, without complications. Endoscopic evaluation suggested that the injected sECM/MC hydrogel remained in the paralyzed vocal folds for up to 8 weeks and augmented volume in the paralyzed left vocal fold in all rabbits of group 3 (*n* = 4). Moreover, group 3 showed straighter vocal folds on the paralyzed side than those of the control group, which were curved and shortened (Fig 1).

High-speed imaging of vocal fold vibration that was induced by intra-tracheal airflow showed regular and symmetrical vocal contact in the sECM/MC hydrogel groups. In addition, the left vocal fold, which was injected with the sECM/MC hydrogel, had a vibration similar to the normal side (Fig 2A). The mean asymmetry index in the sECM/MC hydrogel groups was closer to 1.0 than that of the control group (1.020 ± 0.069, 0.787 ± 0.102, respectively). There was significant difference between two groups (*p* = 0.047 using a Mann-Whitney U test, Fig 2B and 2C).

**Fig 3:**
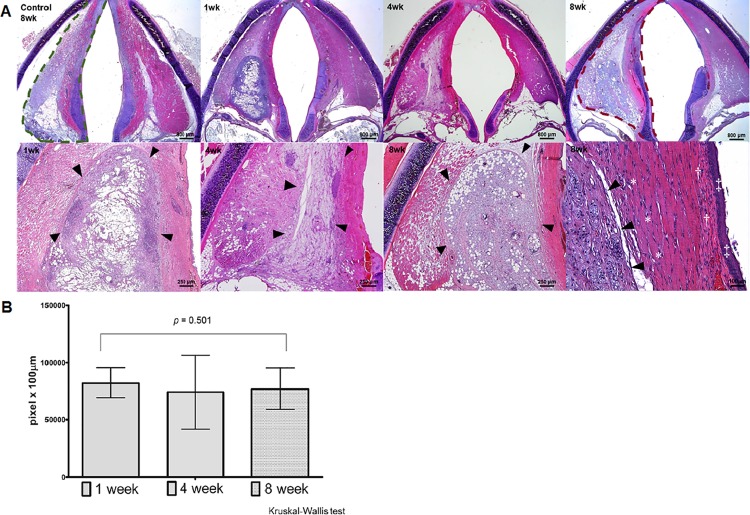
**Standard hematoxylin and eosin (H&E) staining of rabbit larynx after injection laryngoplasty into the left paralyzed vocal fold (A) and the quantitative analysis of remaining volume of sECM/MC hydrogels (B).** (A) Histological examination of the injected biomaterials at 8 weeks post procedure. Area of the laryngeal intrinsic muscle was smaller on the denervated side in the control group (green dotted line) than on the contralateral normal side. In the sECM/MC group, the laryngeal muscle area was compensated for by the injected sECM/MC hydrogel (brown dotted line). The injected sECM/MC hydrogel (arrowhead) induced no significant inflammatory response including neutrophils or lymphocytes aggregation in the surrounding muscle (*), lamina propria (†), or epithelium (‡). (B) Quantitative analysis of remaining sECM/MC hydrogel volume (*p* = 0.501 using Kruskal-Wallis test).

Histological analysis of H&E-stained sections revealed no significant resorption of the injected sECM/MC hydrogel until 8 weeks post injection. The control group showed smaller area of the larynx on the denervated side than on the normal contralateral side at 8 weeks post procedure (Fig 3A, green dotted line). This is likely due to the obvious atrophy of the intrinsic laryngeal muscles. However, in the sECM/MC group, the laryngeal volume was compensated for by the injected sECM/MC hydrogel (Fig 3A, brown dotted line). Quantitative analysis using H&E staining of sequential tissue sections indicated that the median volume of the sECM/MC hydrogel was stably sustained up to 8 weeks post procedure, and there was no volume difference between 1, 4, and 8 week (*p* > 0.05 by Kruskal-Wallis test, Fig 3B).

**Fig 4:**
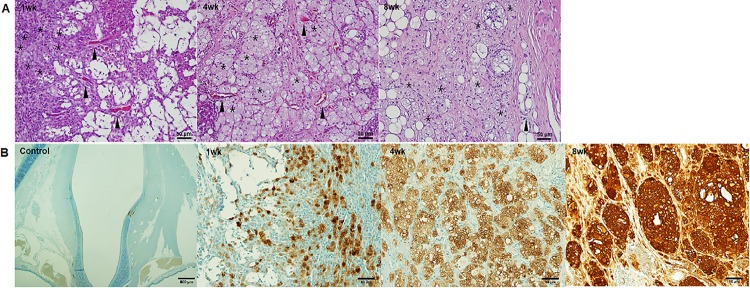
Foamy histiocyte with intracellular fatty lobules and neo-vascularization in the injected sECM/MC hydrogels. (A) Focal capillary ingrowths (arrowhead) into the injection site. Cell aggregations with intracellular fatty lobules in the injection area (asterisk). (B) RAM 11 positive cells (dark brown) are foamy histiocytes, which increased as a function of time.

Focal capillary ingrowth into the injection site (Fig 4A, arrowheads) was observed without any significant inflammatory response, including neutrophil and lymphocytes aggregation in the surrounding muscle, lamina propria, or epithelium (Figs 3A and 4A). Interestingly, cell aggregates with intracellular fatty lobules developed in the area of the sECM/MC hydrogel injection (Fig 4A, asterisk). Additional IHC analysis using RAM 11 suggested that these cells were foamy histiocytes that had phagocytized fatty granules in the injected sECM/MC hydrogel (Fig 4B).

**Fig 5:**
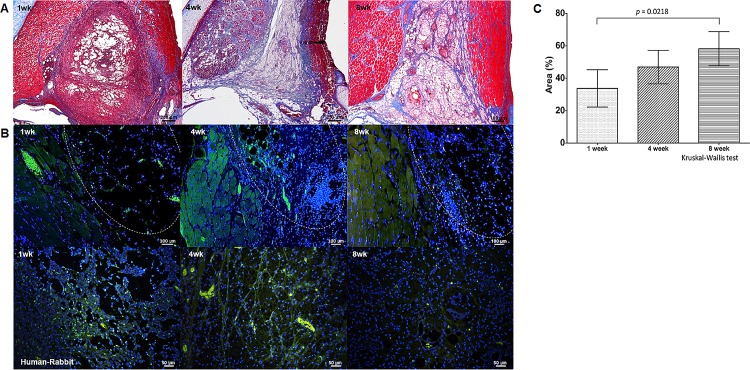
**Collagen fiber stained by MT staining (A), IF for Type I collagen of Human/Rabbits (B) and the quantitative analysis of collagen fiber deposition (area %) in the injected sECM/MC hydrogel (C).** (A) MT staining of collagen (blue) in the injected sECM/MC hydrogel at 1, 4 and 8 weeks post procedure. (B) Type I collagen fibers of the intrinsic vocal muscle and the injected sECM/MC hydrogel (white dotted line) are shown (blue: DAPI, green: collagen type I). (C) The mean percent areas (%) of collagen fibers in the injection area are shown (*p* = 0.0218 by Kruskal-Wallis test).

Collagen fibers were weakly stained with MT in the area of sECM/MC hydrogel injection in group 1 (1 week). However, MT staining in group 2 (4 weeks) and group 3 (8 weeks) indicated increased collagen deposition (Fig 5A). Similar results were observed using quantitative analysis of collagen deposition (Fig 5C). The mean cross-sectional area (%) of collagen fiber at the injection site in group 1 was 33.72 ± 11.48%; in group 2, 46.9 ± 10.36%; and in group 3, 58.23 ± 10.55%. The total collagen deposition at 4 and 8 weeks was significantly higher than that at 1 week (*p* = 0.0218 by Kruskal-Wallis test). The deposition of collagen type I was slightly increased up to 4 weeks post procedure in the injection area as determined using IF (Fig 5B). However, collagen type I was not detected at 8 weeks post procedure (Fig 5B). The formation of elastin fibers or hyaluronic acid was not detected in the area of injection (assessed by VVG and AB staining, S1 Fig).

## Discussion

Injected sECM/MC was stable up to 8 weeks after injection without early resorption, and augmented volume in the paralyzed vocal fold lasted for at least 8 weeks as indicated by endoscopy and histology. In study of asymmetry index, physiologic symmetricity of vocal fold vibration was recovered up to near normal levels (index value = 1) in the sECM/MC-injected groups, whereas vibration in the control group exhibited asymmetricity. These results suggest that injection of sECM/MC hydrogel in paralyzed vocal folds augmented volume and resulted in recovery of vocal fold function. In addition, collagen type I was not detected in the injection area, suggesting excellent biocompatibility without a significant inflammatory response. Though diagnosis of fibrosis is not based solely on excess collagen type I deposition, the presence of collagen type I typically increases significantly during fibrogenesis, in a tissue-context dependent manner.[24, 25]

ECM plays a central role in tissue engineering by preserving tissue volume, providing temporary mechanical functions, and guiding the complex multicellular processes of tissue formation and regeneration.[17, 26, 27] ECM influences biological responses such as cell survival, development, cell shape, migration, and cell polarity by interacting with cellular adhesion molecules, growth regulators, binding proteins, proteolytic enzymes, and enzyme inhibitors.[16, 28] Our hypothesis that injecting sECM/MC would induce vocal tissue regeneration was supported by histological findings. The total collagen fiber content was increased up to 8 weeks post procedure as determined using MT staining. Furthermore, we detected foamy histiocytes that phagocytized fatty granules at the injection site, which suggests that adipose tissue-derived sECM may induce regeneration of fatty components. Previous work has indicated a clear role for adipogenesis during transplantation of adipose tissue-derived sECM.[14–18] Phagocytosis of human fatty materials is a predictable result in the immune-competent rabbit rather than in immune-compromised animals.[29–32] These results are more favorable for vocal fold tissue recovery than our previously published reports using CaHA injection, which induced the formation of abundant giant cells. However, this study is based only on a preliminary animal study with a relatively short time course (8 weeks). Further studies on sECM/MC injections will be needed to corroborate these results for long-term tissue regeneration in paralyzed vocal folds.

We believe the novel sECM/MC hydrogel has several advantages as a material for injection laryngoplasty. First, total medical expense of injection laryngoplasty could be lowered by using sECM/MC hydrogel. sECM/MC can be easily injected in the outpatient clinic under local anesthesia and consequently the medical expense would be much lower compared with that of conventional procedures with fat injection which must be done in operating room under general anesthesia. Second, the biostability of sECM/MC hydrogels could enable injections to be more accurate in quantity and localization than that achieved with conventional fat or CaHA injections. Conventional injection materials for augmentation must be over-injected to compensate for early resorption of the material. Avoiding over-injection would directly improve post injection voice quality. Finally, the straightforward ECM solubilization process and ability to store the sECM/MC hydrogels using refrigeration could permit more frequent use of autologous adipose tissue-derived sECM injections. Considering that liposuction has become one of the most commonly performed aesthetic surgery procedures,[33] autologous fat tissue is easily obtained. If aseptic facilities capable of harvesting and handling adipose tissue are improved, injection of autologous sECM extracts may become more readily available.

We believe there are direct clinical applications for injection laryngoplasty using sECM/MC hydrogels. The material is effective for volume augmentation of paralyzed vocal folds, and the long-term effects are likely due to adipogenesis. These injections use small bore needles and can be administered at outpatient clinics. Finally, the material has excellent biocompatibility and restores vocal fold vibration.

## Conclusions

To the best of our knowledge, this is the first report that uses an injectable form of human adipose tissue-derived sECM/MC hydrogels for vocal fold injection. We propose sECM/MC hydrogels as a highly suitable material for augmentation injection laryngoplasty. Further studies are needed to elucidate the mechanisms by which sECM/MC injections induce tissue regeneration and their long-term effects.

## Supporting Information

S1 Fig Verhoeff-Van Gieson and Alcian blue staining of larynx. Verhoeff-Van Gieson (VVG) and Alcian blue (AB) staining of rabbit larynx after injection laryngoplasty into the left paralyzed vocal fold.
(TIF)
