# Supplementary figures and images for: Human Adipose Tissue Derived Extracellular Matrix and Methylcellulose Hydrogels Augments and Regenerates the Paralyzed Vocal Fold
Source: PLoS One. 2016 Oct 21;11(10):e0165265. doi: 10.1371/journal.pone.0165265 (PMC5074505; doi:10.1371/journal.pone.0165265)

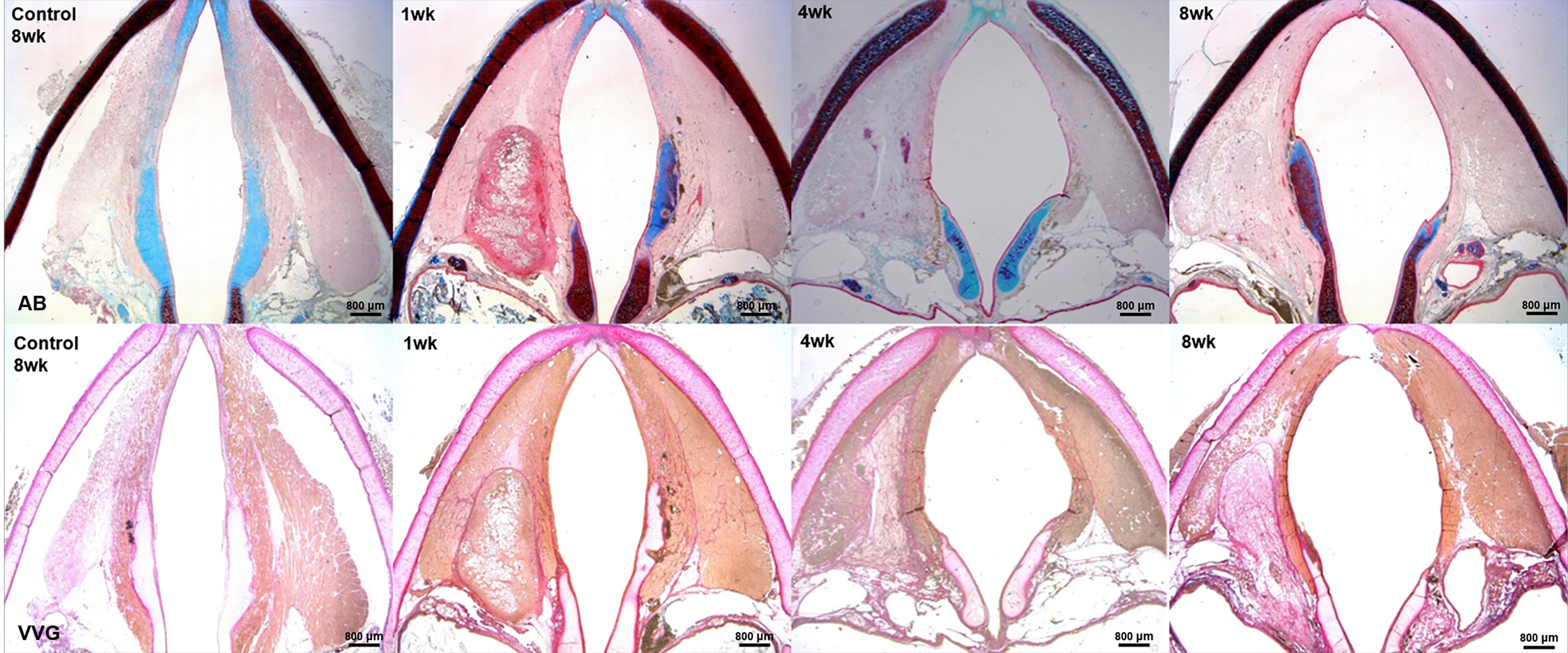

Supplement: S1 Fig — Verhoeff-Van Gieson (VVG) and Alcian blue (AB) staining of rabbit larynx after injection laryngoplasty into the left paralyzed vocal fold. (TIF) [file pone.0165265.s001.tif]
